# Inhibition of tumor necrosis factor alpha reduces the outgrowth of hepatic micrometastasis of colorectal tumors in a mouse model of liver ischemia-reperfusion injury

**DOI:** 10.1186/1423-0127-21-1

**Published:** 2014-01-08

**Authors:** Shu-Fan Jiao, Kai Sun, Xiao-Jing Chen, Xue Zhao, Ning Cai, Yan-Jun Liu, Long-Mei Xu, Xian-Ming Kong, Li-Xin Wei

**Affiliations:** 1 Medical Sciences Research Center, Renji hospital, School of Medicine, Shanghai Jiaotong University, 1630 Dongfang Road, Shanghai 200127, China; 2 Tumor Immunology and Gene Therapy Center, Eastern Hepatobiliary Surgery Hospital, Second Military Medical University, Shanghai, China; 3 Shanghai Pharmaceuticals Holding Co., Ltd, Shanghai, China

**Keywords:** Colorectal cancer, Liver metastases, Ischemia-reperfusion, TNF-α, Enbrel

## Abstract

**Background:**

Patients with colorectal cancer (CRC) often develop liver metastases, in which case surgery is considered the only potentially curative treatment option. However, liver surgery is associated with a risk of ischemia-reperfusion (IR) injury, which is thought to promote the growth of colorectal liver metastases. The influence of IR-induced tumor necrosis factor alpha (TNF-α) elevation in the process still is unknown. To investigate the role of TNF-α in the growth of pre-existing micrometastases in the liver following IR, we used a mouse model of colorectal liver metastases. In this model, mice received IR treatment seven days after intrasplenic injections of colorectal CT26 cells. Prior to IR treatment, either TNF-α blocker Enbrel or low-dose TNF-α, which could inhibit IR-induced TNF-α elevation, was administered by intraperitoneal injection.

**Results:**

Hepatic IR treatment significantly promoted CT26 tumor growth in the liver, but either Enbrel or low-dose TNF-α pretreatment reversed this trend. Further studies showed that the CT26 + IR group prominently increased the levels of ALT and AST, liver necrosis, inflammatory infiltration and the expressions of hepatic IL-6, MMP9 and E-selectin compared to those of CT26 group. Inhibition of TNF-α elevation remarkably attenuated the increases of these liver inflammatory damage indicators and tumor-promoting factors.

**Conclusion:**

These findings suggested that inhibition of TNF-α elevation delayed the IR-enhanced outgrowth of colorectal liver metastases by reducing IR-induced inflammatory damage and the formation of tumor-promoting microenvironments. Both Enbrel and low-dose TNF-α represented the potential therapeutic approaches for the protection of colorectal liver metastatic patients against IR injury-induced growth of liver micrometastases foci.

## Background

Colorectal cancer (CRC) is the third most frequently diagnosed cancer in men and the second most frequently diagnosed cancer in women worldwide [1]. A significant proportion of patients with primary CRC go on to develop metastatic disease, which makes CRC eradication difficult in these patients [2,3]. The liver is the most common site for the metastatic spread of CRC. The development of liver metastases is the major determinant of survival in about 50% of CRC patients [4]. In these cases, surgery is considered the only potentially curative option [5].

Liver surgery entails the occlusion of hepatic blood vessels and as a result is associated with ischemia-reperfusion (IR) injury. After IR injury, the damaged liver tissue becomes infiltrated with inflammatory cells, and the associated release of inflammatory mediators is thought to promote the development of metastatic foci [6]. Indeed, studies in rats have shown that hepatic IR can promote the growth of liver metastasis via the production of E-selectin and matrix metallopeptidase-9 (MMP9) [7,8]. Furthermore, development of IR has been shown to cause a remarkable increase in the serum level of tumor necrosis factor alpha (TNF-α), mainly through release from activated Kupffer cells [9,10]. Other studies have shown TNF-α to further induce cytokines and production of granulocyte colony-stimulating factor, which in turn further enhance Kupffer cell activation and promote neutrophil infiltration in the liver [11,12].

Enbrel (etanercept) is a genetically engineered, soluble, systemic TNF-α blocker that competitively binds to and neutralizes both soluble and transmembrane forms of TNF-α [13,14]. The drug is well tolerated in humans, and is used to treat chronic inflammatory diseases such as rheumatoid arthritis and ankylosing spondylitis [15,16]. Some studies have suggested that pretreatment with low-dose TNF-α can inhibit IR-induced elevations in serum TNF-α level [17,18]. In the light of these findings, in the present study, we aimed to investigate whether Enbrel and low-dose TNF-α pretreatment could prevent IR-enhanced outgrowth of colorectal liver metastases and the underlying mechanism in a mouse model.

## Methods

### Animals

Male wild-type BALB/C mice (age, 10–12 weeks; weight, 25–29 g) were purchased from the Shanghai Experimental Center of the Chinese Science Academy, Shanghai, and housed under pathogen-free conditions. All animal experiments were carried out in accordance with animal experimentation protocols approved by the Animal Care Committee of Shanghai Jiaotong University.

### Carcinoma cell culture and induction of liver metastases in mice

CT26 (a murine colon carcinoma cell line) cells were cultured in Dulbecco’s Modified Eagle Medium (DMEM; GIBCO Life Technologies, Grand Island, New York) containing 10% fetal bovine serum (GIBCO), penicillin (100 U/ml), and streptomycin (100 μg/ml) at 37°C in a humidified atmosphere containing 5% CO_2_. Confluent cultures were harvested by brief trypsinization (0.05% trypsin in 0.02% EDTA), and after centrifugation, single cell suspensions were prepared in physiological saline (10^6^ cells/100 μl). Then, 100 μl of the cell suspension (10^6^ cells) was injected into the parenchyma of the spleens of the animals. After 3 min, the spleens were removed to prevent intrasplenic tumor growth. Animals were reared for a further 5 days to allow sufficient time for liver metastasis to develop.

### Murine model of hepatic IR injury

The mice were anesthetized with pentobarbital (intraperitoneal injection, 50 mg/kg), and partial hepatic IR was induced by clamping the left hepatic artery, portal vein, and bile duct to the left and middle lobes of the liver for 15 min. Surgical procedures were performed under aseptic conditions, and to prevent dehydration, a small amount of saline was left in the abdominal cavity, which was covered with gauze. The animals were kept on a heated table to maintain a body temperature of 37°C.

### Experimental groups

The mice were divided into 5 treatment groups: (1) sham group (n = 5), intraperitoneal injection of saline only, followed by a sham surgical procedure; (2) CT26 group (n = 5), injection of CT26 cells, but no surgery for induction of IR injury; (3) CT26 + IR group (n = 5) injection of CT26 cells followed by surgery for induction of IR injury; (4) CT26 + IR + Enbrel group (n = 5), injection of CT26 cells, surgery for induction of IR injury, and pretreatment with the TNF-α blocker Enbrel (1 mg/kg, i.p. injection 16 h before surgery for IR induction); and (5) CT26 + IR + TNF-α group (n = 5), injection of CT26 cells, surgery for induction of IR injury, and pretreatment with recombinant murine TNF-α (5 μg/kg, i.p. injection 30 min before surgery for IR surgery; PEROTECH, Rocky Hill, NJ).

### Measurement of serum and hepatic TNF-α levels

The mice were euthanized at 0, 30, 90, 180, and 360 min after surgery for IR induction, and the levels of serum TNF-α and hepatic TNF-α in liver homogenates were quantified using a mouse-specific TNF-α ELISA kit (DAKEWE, Shenzhen, China) according to the manufacturer’s instructions.

### Tumor analysis

The mice were euthanized at 7 days following surgery for IR induction. The livers were calculated total tumor volume per liver [volume (mm^3^) = (long diameter × short diameter^2^)/2] and visible tumor numbers per liver. The tumor load of each excised liver was quantified by a hepatic replacement area (HRA) score, which was was the percentage of tumor tissue in the whole hepatic tissue. On 3 nonsequential H&E stained sections per liver, 30 random fields (objective magnification 100×) were selected and were used to calculate the ratio of tumor cells versus normal hepatocytes plus necrotic cells. The average percentage of tumor tissue of all the fields was expressed by HRA.

### Biochemical analysis

The serum levels of alanine aminotransferase (ALT) and aspartate aminotransferase (AST) were examined using a Fuji DRICHEM 55500 V (Fuji Medical System, Tokyo, Japan) according to the manufacturer’s instructions.

### Histopathology analysis

The livers were fixed using 10% neutral-buffered formalin and embedded in paraffin. Sections of the livers were stained with hematoxylin and eosin (H&E) stain and examined for evidence of morphological changes. The livers were examined for signs of tissue necrosis at 360 min after reperfusion by using a myeloperoxidase (MPO) assay kit (Nanjing Jiancheng Bioengineering Co Ltd, Nanjing, China) to measure liver homogenate MPO levels, a marker of neutrophil infiltration.

### Quantitative real-time PCR (qPCR)

The mice were harvested at 360 min after IR. Then total RNA of their liver tissues were isolated and purified using Trizol Reagent (Invitrogen, Carlsbad, CA, USA) and RNase-free DNase (Promega, Madison, WI, USA). Oligo dT18-primers and MMLV reverse transcriptase (Promega) were used to prepared complementary DNA. qPCR were performed using a LightCycler 480 system (Roche Diagnostics, Mannheim, Germany). Gene mRNA expressions were analyzed by specific primers as follows: IL6, Forward-5′-GTCAACTCCATCTGCCCTTC-3′, Reverse-5′-CTTGGTCCTTAGCCACTCCT-3′; MMP9, Forward-5′-CAATCCTTGCAATGTGGATG-3′, Reverse-5′-TAAGGAAGGGGCCCTGTA AT-3′; E-selectin, Forward-5′-CTCCTGCGAAGAAGGATT TGA-3′, Reverse-5′-CCCCTC TTGGACCACACTGA-3′. Endogenous β-actin was used as an internal control to determined the fold change of gene expression, which primer as follows: Forward-5′-AGATGTGGATC AGCAAGC AG-3′, Reverse-5′-GCGCAAGTTAGGTTTTGTCA-3′.

### Statistical analysis

All data are presented as means ± SEM, which are in each case averaged from 3 independent experiments. Observed differences between the treatment groups were analyzed using the Student’s t-test and one-way ANOVA to test for statistical significance; *P* < 0.05 was considered statistically significant. Statistical analyses were performed using GraphPad Prism 5.0 software (Graphpad Software, San Diego, CA).

## Results

### Effects of Enbrel and low-dose TNF-α treatment on IR-induced upregulation of TNF-α level

**Figure 1:**
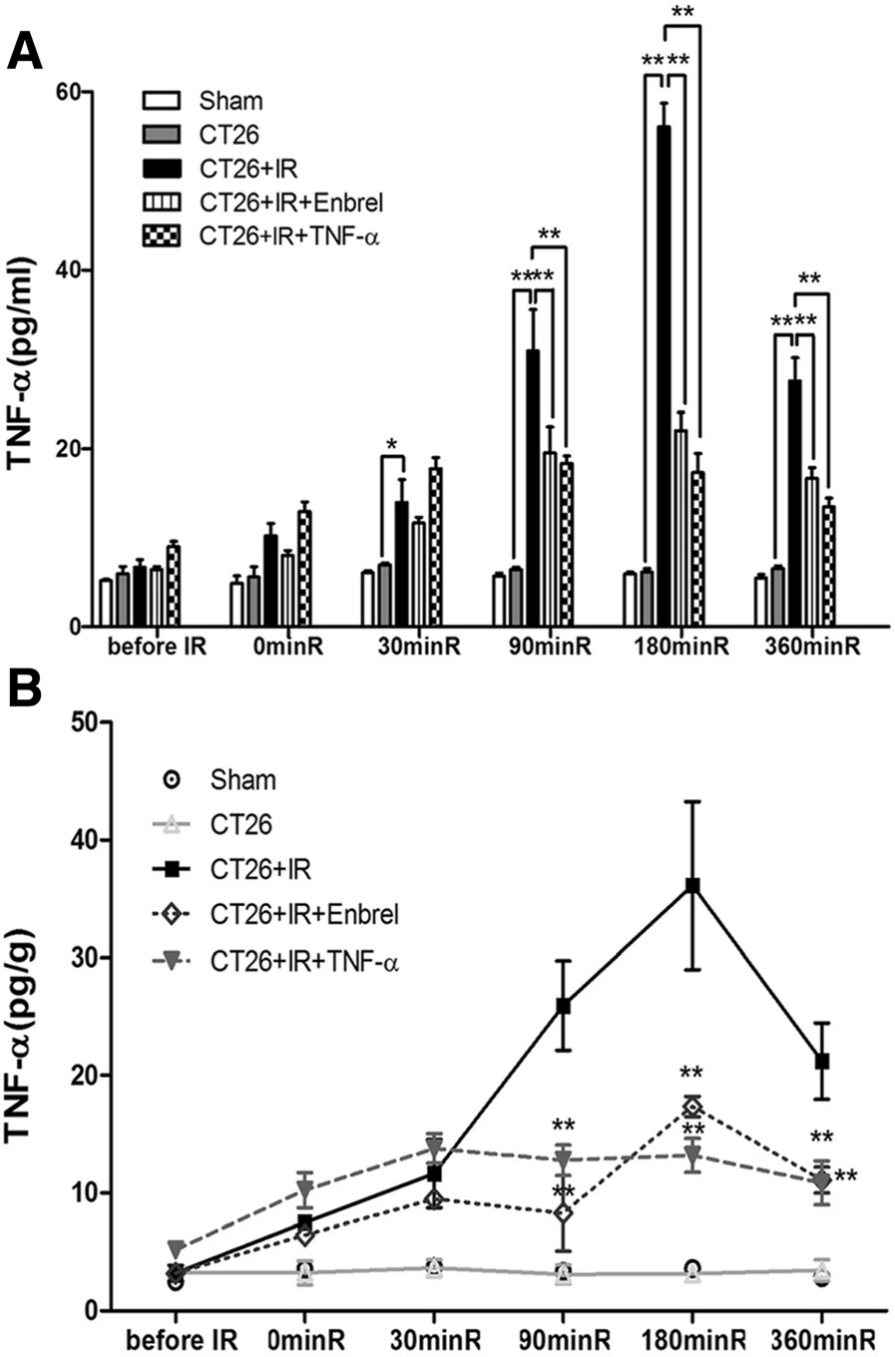
**Effects of Enbrel and low-dose TNF-α pretreatments on TNF-α concentrations in the process of IR.** The mice of each group were sacrificed at indicated times, and the TNF-α levels of **(A)** serum and **(B)** liver homogenates were determined by ELISA. minR: minutes after reperfusion. Data are presented as mean ± SEM. (n = 5; *, P < 0.05; * *, P < 0.01). The data represent three separate experiments.

Compared to the Sham and CT26 only groups, the CT26 + IR group showed significantly elevated serum TNF-α level (Figure 1A) and hepatic TNF-α level (Figure 1B). Peak concentrations of TNF-α were observed at 180 min after reperfusion (Figure 1A, B). Both Enbrel and low-dose TNF-α pretreatment remarkably decreased the serum and hepatic TNF-α levels, at 90 min, 180 min, and 360 min after IR induction (Figure 1A, B).

### Effects of TNF-α inhibition on IR-induced acceleration of tumor growth

**Figure 2:**
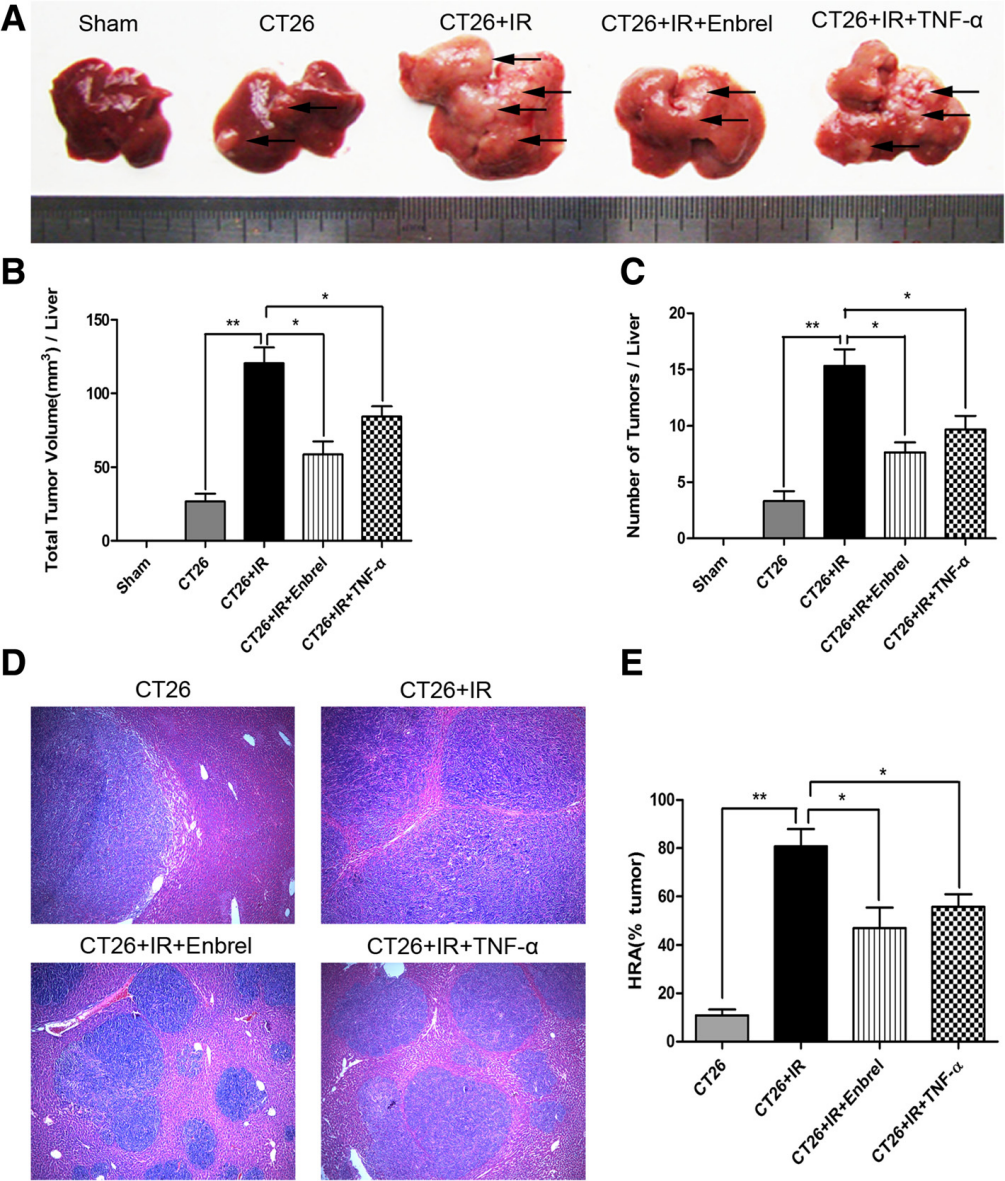
**TNF-α inhibition restrained IR-enhanced colorectal tumor growth in the liver.** The mice were sacrificed at seven days after IR, and their livers were harvested to estimated tumor growth. **(A)** Tumor load (The arrows indicated tumors), **(B)** total tumor volume, mm^3^ = (long diameter × short diameter^2^)/2 **(C)** tumor number (per liver of each animal were counted). **(D)** H&E stained histopathological sections (magnification × 50) and **(E)** percentages of hepatic replacement area were shown. Data are presented as mean ± SEM. (n = 5; *, *P* < 0.05; * *, *P* < 0.01). The data represent three separate experiments.

IR treatment led to a significant increase in tumor growth; however, both Enbrel and low-dose TNF-α pretreatment tended to attenuate this increase (Figure 2A). Compared to the CT26 + IR group, the CT26 + IR + Enbrel group showed a 2-fold lower total tumor volume (120.5 ± 18.7 mm^3^ vs. 58.7 ± 15.5 mm^3^), and a 2-fold lower tumor number (15.3 ± 2.5 vs. 7.7 ± 1.5) (Figure 2B, C). Low-dose TNF-α pretreatment also showed similar effects on tumor growth. Furthermore, the percentage of liver tissue replaced by tumor cells was significantly lower in CT26 + IR + Enbrel and CT26 + IR + TNF-α pretreatment groups compared to the CT26 + IR group (Figure 2D, E). Taken together, these observations suggest that both Enbrel and low-dose TNF-α pretreatment effectively reduced IR-induced growth of colorectal liver metastases.

### Effects of TNF-α-inhibition on IR-induced liver enzymes

**Figure 3:**
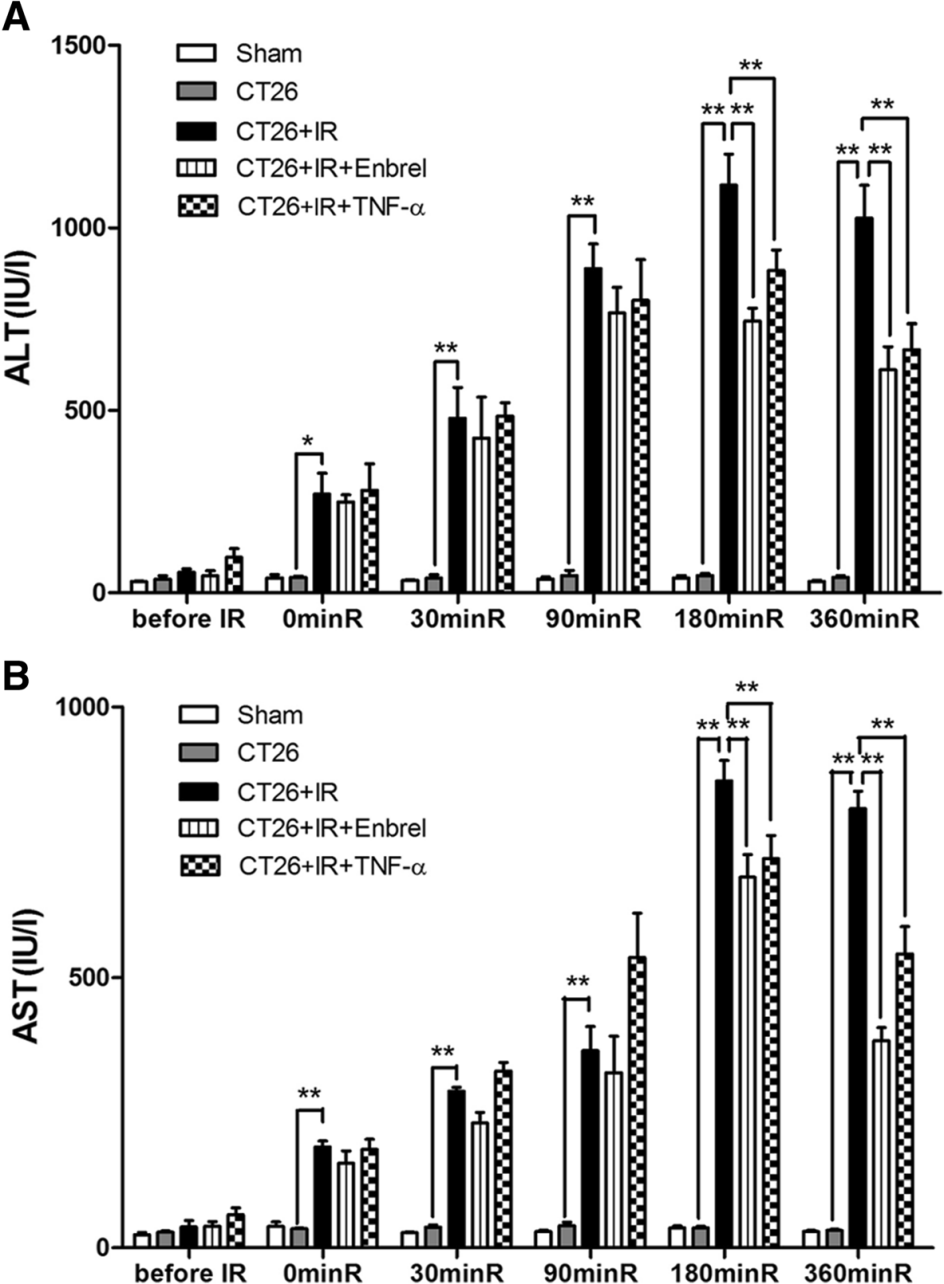
**Effects of TNF-α inhibition on serum levels of ALT and AST in the process of IR.** The mice of each group were sacrificed at 6 h after IR and were detected the serum concentrations of **(A)** ALT and **(B)** AST were examined using a Fuji DRICHEM 55500 V. Data are presented as mean ± SEM. (n = 5; *, *P* < 0.05; * *, *P* < 0.01). The data represent three separate experiments.

Compared to the CT26 group, the CT26 + IR group showed significantly elevated serum levels of ALT (Figure 3A) and AST (Figure 3B). Serum ALT and AST levels were significantly decreased at 180 min and 360 min after reperfusion (Figure 3A, B) in both the Enbrel and low-dose TNF-α pretreatment groups relative to the CT26 + IR group. These data suggest that both Enbrel and low-dose TNF-α pretreatment prevented IR-induced liver injury to some extent.

### Effects of TNF-α-inhibition on IR-induced inflammatory response and hepatic injury

**Figure 4:**
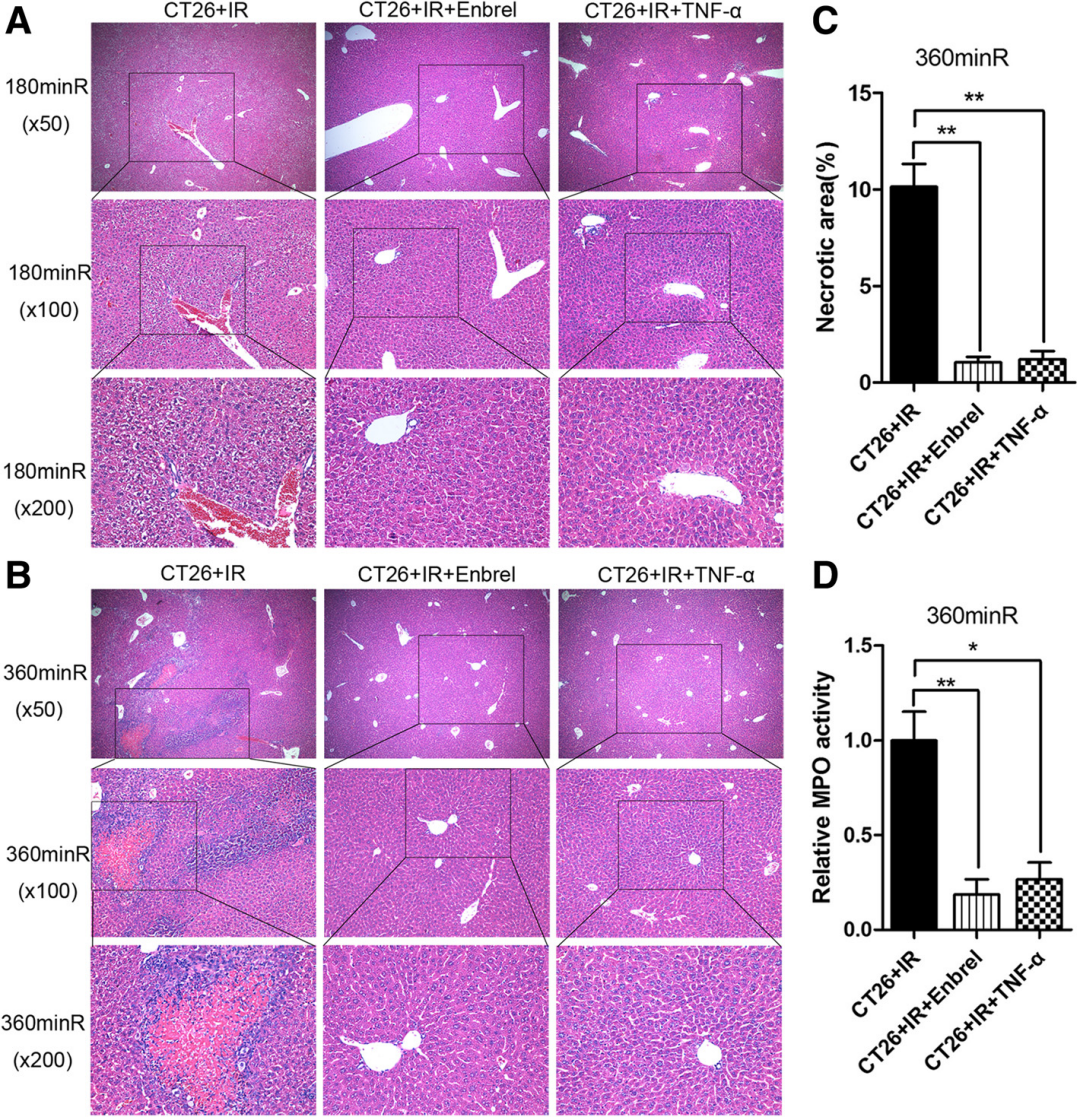
**Inhibition of TNF-α suppressed IR-induced liver injury.** The mice of each group were sacrificed at indicated times after IR. H&E stained histopathological sections at **(A)** 180 min, **(B)** 360 min. **(C)** Percentages of necrotic area and **(D)** relative MPO activity of liver homogenate at 360 min were shown. Data are presented as mean ± SEM. (n = 5; *, *P* < 0.05; * *, *P* < 0.01). The data represent three separate experiments.

Microscopy examination revealed that compared to the liver tissues of the CT26 and sham groups, the liver tissues of the CT26 + IR group had significant cytoplasmic vacuolization at 180 min after IR (Figure 4A), and extensive cell necrosis with marked inflammatory cell infiltration at 360 min after IR (Figure 4B). However, liver tissue necrosis was significantly reduced by Enbrel and low-dose TNF-α pretreatment (Figure 4C). MPO concentrations of liver homogenate significantly increased in the CT26 + IR group, and remarkably decreased in both the Enbrel and low-dose TNF-α pretreatment groups (Figure 4D). These observations suggest that the TNF-α-inhibiting pretreatments reduce IR-induced hepatic inflammation and necrosis.

### Effects of Enbrel and low-dose TNF-α treatment on IR-induced mRNA expressions of IL-6, MMP9 and E-selectin

**Figure 5:**
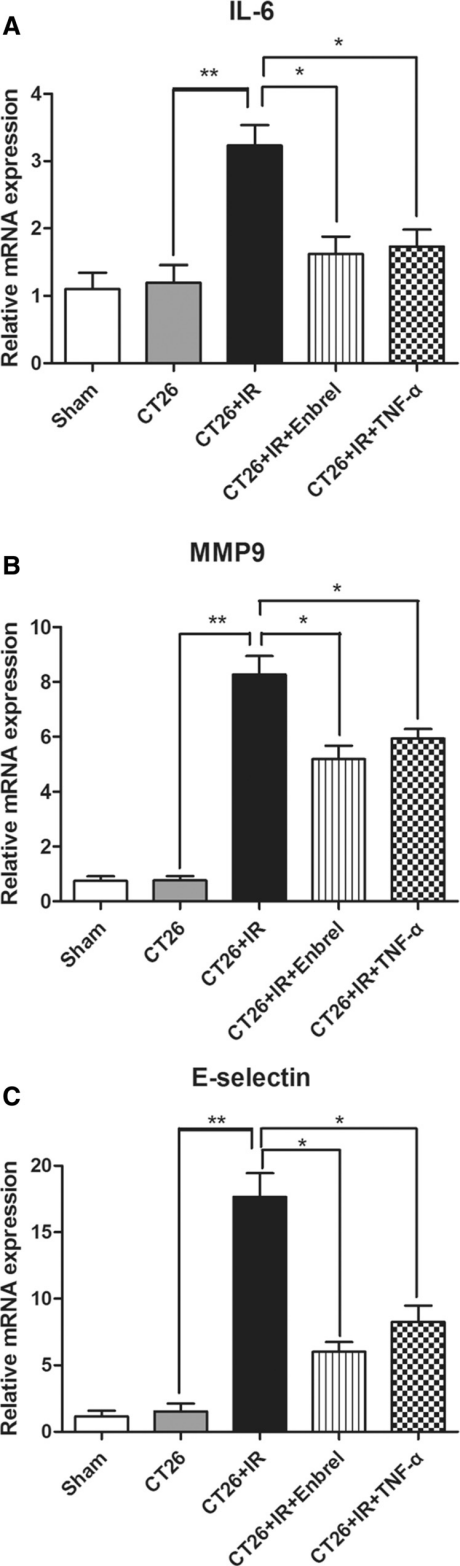
**Inhibition of TNF-α reduced IR-induced liver mRNA expressions of IL-6, MMP9 and E-selectin.** The liver of each mice were harvested at 360 min after IR and their mRNA expressions of **(A)** IL-6, **(B)** MMP9 and **(C)** E-selectin were detected by qPCR. Data are presented as mean ± SEM. (n = 5; *, *P* < 0.05; * *, *P* < 0.01). The data represent three separate experiments.

RT-PCR results showed that liver tissues mRNA expressions of IL-6 (Figure 5A), MMP9 (Figure 5B) and E-selectin (Figure 5C), significantly increased in CT26 + IR group compared with CT26 and sham groups. Both the Enbrel and low-dose TNF-α pretreatment groups remarkably decreased the level of liver tissues mRNA expressions of IL-6, MMP9 and E-selectin compared to CT26 + IR group. These results suggest that both Enbrel and low-dose TNF-α pretreatment significantly reduced IR-induced elevated of IL-6, MMP9 and E-selectin mRNA expressions in the IR liver.

## Discussion

The findings of this study demonstrated that both Enbrel and low-dose TNF-α pretreatment before IR, remarkably decreased serum and hepatic TNF-α levels, reduced tumor growth, decreased serum ALT and AST levels, reduced hepatic tissue injury and cytoplasmic vacuolization of cells, and reduced hepatic cellular necrosis and infiltration of inflammatory cells. These findings suggest that TNF-α might play an important role in IR-accelerated outgrowth of colorectal liver metastases, through the production of an inflammatory response and a microenvironment that is conducive to tumor growth.

A previous study reported that surgical resection for liver metastases of CRC often leads to IR injury [7]. Balkwill et al. reported that TNF-α significantly increased following hepatic IR, an effect mediated in both the early and late phases of liver injury [19]. Some studies have demonstrated that hepatic IR could promote outgrowth of liver metastases of CRC through the increased growth of pre-existing hepatic micrometastases [6,8]. However, the underlying mechanism is not entirely clear; therefore, to elucidate the mechanism, we established a mouse model of colorectal liver metastases as described previously. Following liver IR in this model, we observed that compared to the CT26 group, the CT26 + IR group showed significantly elevated TNF-α levels whereas the CT26 + IR + Enbrel and CT26 + IR + TNF-α groups showed remarkably decreased TNF-α levels at 180 min and 360 min after IR. This result supports the findings of a previous study that showed that low-dose TNF-α pretreatment before IR, reduced serum level of TNF-α and attenuated liver injury [17].

To explore the effect of TNF-α on IR-induced growth of colorectal liver metastases, we examined tumor growth in the excised livers. Our findings showed that compared to the CT26 group, the CT26 + IR group showed significantly increased tumor load whereas the CT26 + IR + Enbrel and CT26 + IR + TNF-α groups showed markedly reduced tumor loads. These results indicated that TNF-α might play a role in IR-induced growth of pre-existing colorectal liver metastases.

Studies in rodent models have shown that pretreatment with an anti-TNF-α antibody, or low doses of TNF-α and pentoxifylline, a methylxanthine inhibitor of TNF-α, prior to hepatic IR, can significantly reduce hepatic injury [17,20-22]. In order to investigate the protective effect of TNF-α inhibition, we examined serum ALT and AST levels at 0, 30, 90, 180, and 360 min after liver IR. We found that compared to the CT26 group, the CT26 + IR group showed significantly increased serum ALT and AST concentrations. The Enbrel and low-dose TNF-α pretreatment groups showed markedly decreased serum levels of ALT and AST at 180 min and 360 min following liver IR.

Several studies have demonstrated that IR-related liver injury results from an severe inflammatory response involving the release of TNF-α by Kupffer cells, which alone can further intensify the inflammation reaction via the production of vascular adhesion molecules and neutrophil-attracting chemokines [23-27]. Our study included a histopathological analysis to determine microscopic changes in the liver following IR. The analysis revealed that compared to the CT26 group, the CT26 + IR group showed markedly increased cytoplasmic vacuolization, inflammatory cell infiltration, and hepatic cellular necrosis, whereas the CT26 + IR + Enbrel and CT26 + IR + TNF-α groups showed significantly reduced cytoplasmic vacuolization, inflammatory cell infiltration, and hepatic cellular necrosis. These results indicated that inhibition of TNF-α could protect against IR-induced hepatic tissue damage through a decrease in TNF-α and the inflammatory response. Indeed, inflammation plays a crucial role in promoting tumor development and metastasis [28,29], and there is much evidence to suggest that TNF-α is a key pro-inflammatory cytokine involved in tumorigenesis [19]. Previous studies reported that the upregulation of IL-6 [17,21], MMP-9 [30,31] and E-selectin [7,32,33] levels followed by IR were directly involved in hepatic damage. Meanwhile, IL-6 and MMP9 have been shown to promote the growth of colon cancer [8,34,35]. Our current study observed that both Enbrel and low-dose TNF-α pretreatments before IR markedly reduced the mRNA expressions of tumor promoting factors, IL-6 MMP-9 and E-selectin in IR liver.

Taken together, our results suggest inhibition of TNF-α in the tumor microenvironment through a reduction in inflammatory cell infiltration, following liver IR, and our findings are consistent with that of previous research, for example, the inhibition or neutralization of TNF-α reduces the infiltration of inflammatory cells into hepatic tissue, and reduces liver IR injury [9,22]. Studies have shown that inflammatory mediators can disrupt the extracellular matrix and cause tissue remodeling that allows tumor cell invasion [36], which could promote tumor cell proliferation, survival, invasion, chemoresistance, and angiogenesis [37,38], and lead to the DNA histone methylation, eventually leading to lead to silencing of tumor suppressor loci [39-42]. Achyut BR and his colleagues found that deletion of the TGF-β receptor2 gene in stromal fibroblasts induced inflammation and severely damaged DNA, and contributed to the development of invasive squamous cell carcinoma [43].

Taken together our findings suggest that TNF-α could up-regulate the inflammatory response following IR, and possibly produce a microenvironment that promotes tumor growth. Other research supports this, for example, in a TNF-α KO mouse model, hepatic IR injury was attenuated. This study also demonstrated that JNK-1 and NF-κB are activated in both the early and late inflammatory phases of hepatic IR injury, and that TNF-α is main agent for triggering these 2 pathways [22]. Giannandrea M and his colleague showed that TNF-α causes liver injury, but not by a direct cytotoxic effect, rather indirectly by acting as a multiplier of Kupffer cell activation on hepatocytes [44]. Our current study provides further insight into the effect of TNF-α on IR-induced outgrowth of colorectal liver metastases, and also identifies TNF-α as a potential new treatment target, which may eventually lead to a better prognosis for patients undergoing resection for colorectal liver metastases. Finally, while considering the effects of TNF-α in liver injury, tumor promotion and as possible protective treatment for liver IR [45-48], Inflammation microenvironment has the promotive effect in tumor development [49]. The suppressive effect of TNF-α inhibition on IR-accelerated tumor growth may be mediated by attenuating TNF-α -dependent inflammation. One cannot ignore the importance of TNF-α in liver regeneration, which involves NF-κB and p38 [50,51]. Further studies are necessary to provide a detailed mechanism of the potential protective effects of TNF-α inhibition against the growth of liver metastases induced by IR injury.

## Conclusion

In conclusion, our results demonstrated that TNF-α plays an important role in IR-induced outgrowth of colorectal liver metastases by enhancing inflammatory cell infiltration and the formation of the microenvironment that facilitates tumor progression. The finding that pretreatment with both Enbrel and low-dose TNF-α prior to IR protects against liver injury and prevents the growth of liver metastases suggests that these treatments may have the potential for protecting patients undergoing resection for colorectal liver metastases.

## Competing interests

The authors have declared that no competing interests exist.

## Authors’ contributions

SFJ and KS contributed equally to this work. SFJ, KS, LXW, XMK and YJL participated in the design and performance of this study. SFJ and XJC carried out the mice experiment. XZ carried out cell culture. SFJ and NC carried out Biochemical analysis and ELISA experiment. SFJ and LMX carried out HE experiment. LXW and XMK conceived of the study and participated in the design and coordination. The manuscript was drafted by SFJ and KS, and reviewed by all authors. All authors approved the final version of the manuscript to be published.
